# Effects of age and leg length upon central loop of the Gastrocnemius-soleus H-reflex latency

**DOI:** 10.1186/1471-2377-4-11

**Published:** 2004-09-03

**Authors:** Shahram Sadeghi, Mohammadrezaalavian Ghavanini, Alireza Ashraf, Peyman Jafari

**Affiliations:** 1 Pain research group, multidisciplinary pain clinic of iran jahad danesh gahi, no 31, karimkhan zand ave. shahid hosseini alley, Tehran, Iran; 2 Department of physical medicine and rehabilitaton, shiraz medical school, zand avenue, Shiraz, Iran; 3 Department of biostatistics, shiraz medical school, zand avenue, Shiraz, Iran

**Keywords:** central loop of gastrocnemius-soleus H-reflex latency, Central S1 loop latency, sacral radiculopathy

## Abstract

**Background:**

central loop of the gastrocnemius-soleus H-reflex latency (T_c_) that looks promising in the diagnosis of S1 radiculopathy; has been investigated in a few studies and only two of them have focused on the constitutional factors affecting it. Although leg length has been shown to contribute to the T_c_, the role of age is controversial. More confusing, none of the previously performed studies have used strict criteria to rule out subclinical neuropathy, so the results could be misleading. This study has been performed to determine the influence of leg length and age on T_c _among a carefully selected group of healthy volunteers.

**Methods:**

after screening forty six volunteers by taking history, physical examination and a brief electrophysiologic study; forty of them were selected to enroll into the study. T_c _was obtained in all the study subjects and leg length and age were recorded for correlational analyses.

**Results:**

this group was consisted of 26 males (65%) and 14 females (35%) with the age range of 19–65 years (Mean ± SD: 37 ± 10.7) and leg length range of 29.5–43 centimeters (36.4 ± 3.4).

Mean ± SD for T_c _was 6.78 ± 0.3. We found a significant correlation between T_c _and leg length (p value= 0.003, r = 0.49 and confidence interval 95% = 0.59–0.88), no significant correlation was found between age and T_c _(p value= 0.48, r = 0.11), also we obtained the regression equation as: T_c _= 0.04L + 5.28

**Conclusions:**

in contrast to leg length, age was not correlated with T_c_. Future studies are required to delineate other contributing factors to T_c_.

## Background

The H-Reflex evaluates S_1 _radiculopathy [1]. The measured latency, however, is neither specific nor sensitive for S_1 _spinal nerve disease, as it traverses a long pathway. Pease et al [2] were the first who described the central loop of the gastrocnemius-soleus H-Reflex latency (Central S_1 _loop latency or T_c_) and suggested it might be promising in the diagnosis of S1 radiculopathy [1,2].

Unfortunately, T_c _has been the subject of few studies, and as far as we know, only 5 articles [2-6] have been published on this issue so far. Among them, two have specifically evaluated the constitutional factors contributing to T_c_. Leg length has been shown to have a significant effect on T_c_. It is controversial whether age entails a similar effect. Wang et al [5] found a direct correlation between age and Tc. This observation was not confirmed by Ghavanini et al in an independent study [6].

The current study has been performed to determine the influence of leg length and age on T_c_.

## Methods

We enrolled 46 volunteers to this study after obtaining informed consent. Following a standard history taking, all of them underwent physical examination and a brief electrophysiologic evaluation [7] to rule out asymptomatic polyneuropathy, including determination of: right peroneal nerve conduction velocity (PNCV), distal motor latency of right deep peroneal nerve (PDML) and standard gastrocnemius-soleus H reflex latency (T_p_). We defined our exclusion criteria as: history of sacral radiculopathy or diabetes mellitus or any other disease with potential to cause neuropathy, any abnormality in neurological or musculoskeletal physical examination, or any of the following findings: PNCV less than 40 m/s, PDML more than 5 ms or prolonged T_p _(according to Braddom and Johnson's study [8].

Since we were supposed to rule out subclinical peripheral neuropathy and one component of the related electrodiagnostic study was measuring the distal motor latency for the deep peroneal nerve, the temperature at the dorsum of the foot was kept almost at 32°Celsius.

The leg length of each person was measured as the distance from middle of the midpopliteal crease to the point at the most proximal part of the medial malleolus, in centimeters.

Subject's age to the nearest year was also recorded.

For obtaining T_c_, we used DANTEC 2000 c equipment, the sensitivity, sweep, and filter were set at: 0.2–1^mv/div^, 5^ms/div^, and 2–10,000^Hz ^respectively. The technique was the same as described in the Literature [1,2]. Briefly: the volunteers lied prone on the examining table with the feet off the edge of the plinth. The E_1 _was placed at the middle of the line connecting midpoint of popliteal crease to the point at the most proximal part of the medial malleolus, and the E_2 _over the Achilles tendon (both were surface electrodes). The ground electrode was posed proximal to E_1 _and a disc electrode (anode) was placed on the anterior superior iliac spine. Then we inserted a monopolar 70^mm ^needle (cathode) at a point 1^cm ^medial to the posterior superior iliac spine, perpendicular to the frontal plane, and retracted it just a little after reaching the sacrum. Stimulus duration of 1 ms at 0.5 HZ was then applied while increasing current intensity to obtain both H and M waves simultaneously. M wave is the earlier wave and H is the later one. The interpeak latency was measured in milliseconds (ms) and recorded as T_c_. This measurement was only performed on the right lower extremity.

Descriptive statistics were applied to depict Mean ± SD of age, leg length and Tc. The independent effect of leg length and age on T_c _was assessed by multiple regression model. The analyses were performed using SPSS 10.0 software. Kolmogrov-Smirnov test was used for evaluating the normal distribution of the variables.

## Results

From 46 subjects who volunteered to participate in this study; five cases were excluded after history taking and physical examination (two because of history of sacral radiculopathy, two because of diabetes mellitus and one because of asymmetry in ankle reflexes) and one case after electrophysiologic evaluation; thus we completed the study with 40 subjects. Subjects' characteristics are shown in table 1.

**Table 1:** subjects' characteristics

Number of subjects	40 (26 males, 14 females)
Age range (years)	19–65
Leg length (centimeters)	29.5–43
T_c _± SD	6.78 ± 0.3

The group consisted of 26 males (65%) and 14 females (35%). Kolmogrov-Smirnov test showed normal distribution of the variables.

You are provided with the information below: (Mean ± SD) Age: 37.0 ± 10.7 years (range: 19–65); leg length: 36.4 ± 3.4 cm (range: 29.5–43); Tc= 6.78 ± 0.3 There was a significant correlation between T_c _and leg length (P value = 0.003, r= 0.49, CI 95% = 0.59–0.88).

There was no correlation between T_c _and age (p value = 0.48, r = 0.11) We also found this regression equation: T_c _= 0.04L + 5.28 (L is leg length in centimeters, T_c _is represented in milliseconds.)

## Discussion

In this study we found a significant correlation between leg length and Tc, but we were unable to show such a relation between age and T_c_.

Pease et al were the first, studied T_c _[2,4], and reported Mean ± SD of 7 ± 0.3^ms ^which is very close to our results (Tc= 6.78 ± 0.3). They didn't specifically consider the leg length, age or any other potential confounding variables to T_c_.

Zhu et al [3] evaluated 60 persons and reported Mean T_c_: 6.8 ms and its SD: 0.33 ms, again close to our results. They also reported that T_c _and person's height were correlated but didn't study any correlation between age and T_c_.

Wang et al [5] evaluated 40 persons and found this regression equation:

T_c _= 0.02A + 0.003H + 0.92 (H: Height and A: Age), and stated that age is a contributing factor on T_c_.

Another research was performed by Ghavanini et al [6], in which 39 subjects were evaluated. The reported T_c _± SD was 6.9 ± 0.4; two regression equations were also suggested: T_c _= 0.097Tp + 4.045 and Tc = 0.051L + 4.92 (L=leg length in centimeters); results are close to ours, and age was not found to affect T_c_.

A summary of the above data plus detailed demographic data are provided in the table 2.

**Table 2:** comparing related studies

	Subjects' characteristics				Tc		
	
	Group size (persons)	Mean age (yr)	Mean L (cm)	Mean H(cm)	*Mean*	*SD*	Correlation (r) with A-H-L
Present study	40	37.0	36.4 M:38.1 F:33.2	?	6.78	0.3	No-?-0.49
Pease et al [2]	20	?	?	?	7.0	0.3	?-?-?
Zhu et al [3]	60	43	?	169	6.8	0.33	No-0.54-?
Wang et al [5]*	40	?	?	?	?	?	?-?-?
Ghavanini et al [6]**	39	41	M:39.8 F:37.0	M:172.2 F:159.5	6.9	0.4	No-0.56–0.62

A: age (yr); L: leg length (cm); H: height (cm); Tc: central loop of the H-reflex latency (ms); M: male; F: female; No: no correlation was found; ?: not reported

*: suggested a regression equation: Tc = 0.02A+0.03H+0.92

**: Suggested two regression equations: Tc = 0.051L+4.928; Tc = 0.097Tp+4.04

## Limitations

In this study we focused on age and leg length as potential contributing factors on the T_c_. we didn't control, randomize or observe other possible confounding (contributing) factors with potential to affect this parameter.

We observed a significant correlation between leg length and T_c _(P value = 0.003, r= 0.49, CI 95% = 0.59–0.88), that is compatible to a previous published work [3] (r = 0.54, p value less than 0.01).

Had we found any association between T_c _and age, the question might have been raised that subclinical neuropathy of old age could have been contributive; obviously, this is not the case in our study.

Although F-wave has been used to evaluate the possibility of proximal neuropathy; it was not measured in this study. Alternatively, we measured H-reflex latency to exclude proximal neuropathy [11].

It should be emphasized that noninvasive methodologies for the diagnosis of subclinical S1 radiculopathy are now available [12]. It is also acceptable to stimulate the S1 spinal nerve at the S1 foramen by magnet, instead of deep tissue needling; nevertheless, we used more popular techniques for this study.

## Conclusions

We found that between age and leg length, only the latter can affect T_c_. It may be reasonable to consider leg length for calculating T_c _and to "narrow" the normal limits.

Further studies with larger sample sizes are required for detecting other contributing factors and standardizing T_c _according to leg length.

## Competing interests

None declared.

## Abbreviations

T_c_: central loop of the gastrocnemius-soleus H-Reflex latency

T_p_: gastrocnemius-soleus H-Reflex latency

PNCV: right peroneal nerve conduction velocity

PDML: right peroneal nerve distal motor latency

## Authors' contribution

SS: examining the cases, calculation of TC, writing the paper.

MRAG: suggesting the research, supervision and helping with calculation of TC.

AA: examining the cases, calculation of TC

PJ: statistical consultant (data analysis)

## Pre-publication history

The pre-publication history for this paper can be accessed here:


